# Myeloid/lymphoid neoplasm with *FLT3* gene fusion: report of a case with a novel t(5;13)(q13;q12) *SSBP2::FLT3* fusion

**DOI:** 10.1007/s12308-026-00702-9

**Published:** 2026-05-22

**Authors:** James R. Cook, Caroline Astbury, David S. Bosler, John C. Molina, Heesun J. Rogers, Ronald M. Sobecks, Anna B. Owczarczyk

**Affiliations:** 1 Department of Pathology and Laboratory Medicine, Diagnostics Institute, Cleveland Clinic, 9500 Euclid Avenue, Cleveland, OH USA; 2 Department of Hematology and Medical Oncology, Cleveland Clinic Taussig Cancer Institute, Cleveland Clinic, 9500 Euclid Avenue, Cleveland, OH USA

**Keywords:** FLT3 fusion, Myeloid/lymphoid neoplasm with eosinophilia, RNA-NGS

## Abstract

Myeloid/lymphoid neoplasms with eosinophilia and tyrosine kinase gene fusions (M/LN-eo-TK) are diverse, with most cases showing a combination of lymphoblastic leukemia/lymphoma and myeloproliferative features. Various tyrosine kinase fusion genes have been identified, including FLT3 fusions. RNA based next generation sequencing studies offer an effective approach to detect such pathogenic fusion genes, including the novel SSBP2::FLT3 fusion described here. Timely identification of this fusion gene led to targeted management of our patient, who achieved complete resolution of his FDG-avid lymphadenopathy only after the addition of gilteritinib. Two years after successful bone marrow transplant, the patient remains in complete remission. Clinicians and pathologists should have a low index of suspicion for a M/LN-eo-TK when evaluating patients with and without unexplained eosinophilia in the context of extramedullary lymphoid or myeloid disease or unusual myeloproliferative disorders.

## Introduction

The myeloid/lymphoid neoplasms with eosinophilia and tyrosine kinase gene fusions (M/LN-eo-TK) are a group of neoplasms characterized by tyrosine kinase gene fusions in bone marrow stem cells that are capable of differentiating into myeloid and/or lymphoid proliferations [1–3]. This diagnostic category was first recognized in the 4th edition (2008) WHO classification, which included M/LN-eo with *PDGFRA*, *PDGFRB*, or *FGFR1* rearrangements [4]. Patients typically present with bone marrow and peripheral blood involvement by a chronic myeloid neoplasm with eosinophilia that may have either purely myeloproliferative features or a combination of myeloproliferative and myelodysplastic (MPN/MDS) changes, sometimes resembling chronic myelomonocytic leukemia. Patients may also present with blast phase disease which may be acute myeloid leukemia (or myeloid sarcoma), B lymphoblastic or T lymphoblastic leukemia. Complex clinical presentations, including patients with chronic myeloid disease in the bone marrow and lymphoblastic lymphoma at extramedullary sites also occur.

Since the original description of M/LN-eo with *PDGFRA*, *PDGFRB*, or *FGFR1* rearrangements, additional tyrosine kinase fusion genes have been identified. In the revised 4th edition (2016) WHO classification [5], M/LN-eo with *PCM::JAK2* was recognized as a provisional entity. In the International Consensus Classification [6] and 5th edition WHO classifications [7], M/LN-eo with *JAK2* rearrangements, *ETV6::ABL1* rearrangement, and *FLT3* rearrangements are now recognized as distinct entities. M/LN-eo with *FLT3* rearrangements, like other entities in this category, typically present with a bone marrow based myeloid neoplasm in chronic phase that may resemble an MPN, MPN/MDS, or MDS while extramedullary disease, if present, may include myeloid sarcoma, T lymphoblastic lymphoma, or biphenotypic T/myeloid acute leukemia [8, 9]. The most common fusion partner reported to date is *ETV6* (12p13) [2, 3, 10, 11]. In this report, we describe a patient with M/LN-eo-TK with a novel *SSBP2::FLT3* fusion.

## Case presentation

**Fig. 1:**
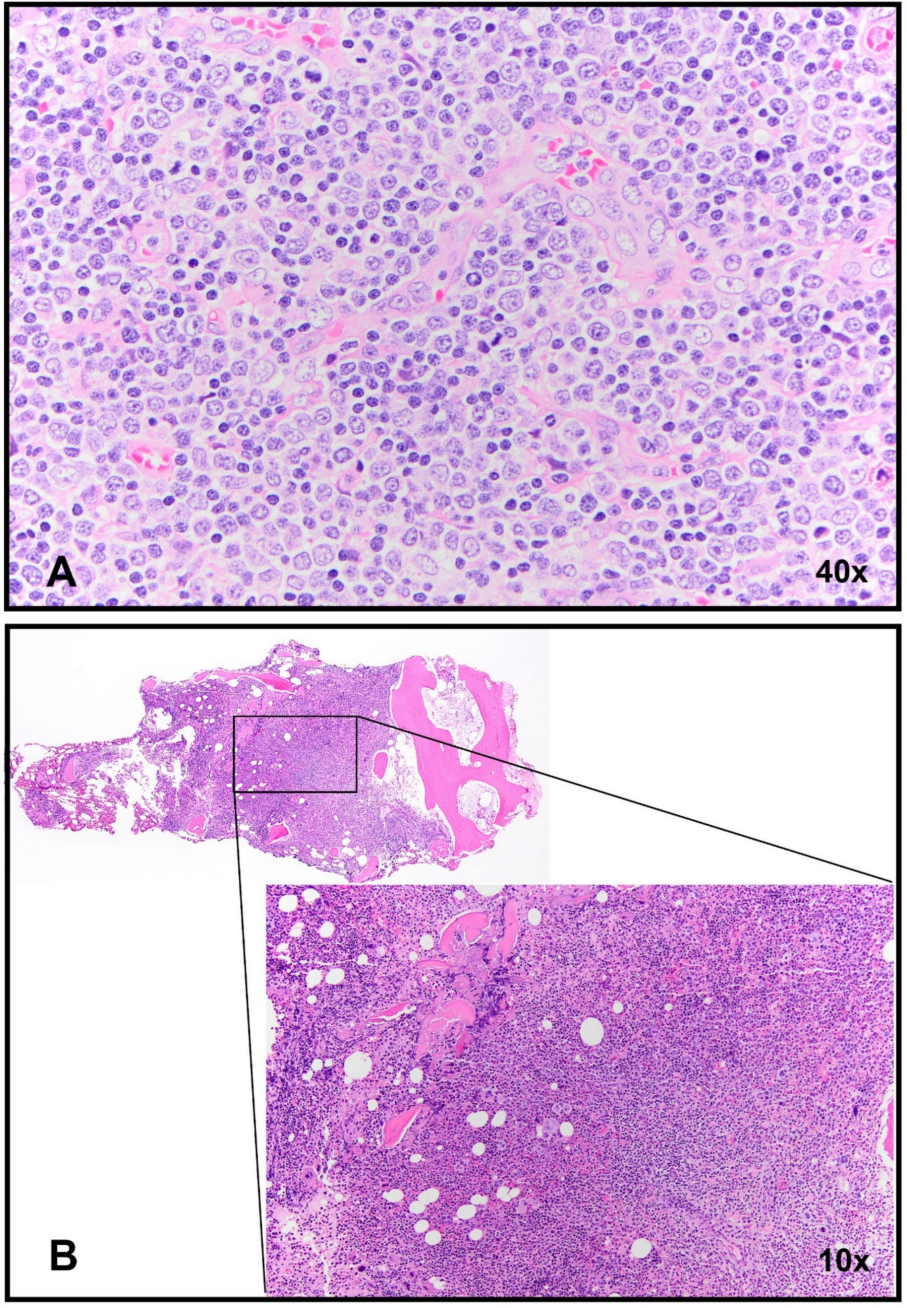
**A** The right groin lymph node shows diffuse infiltration by small to intermediate sized cells with fine chromatin and scant cytoplasm. **B** The bone marrow biopsy is hypercellular with trilineage hematopoiesis and a myeloid predominance

A 37-year-old man presented with a persistent sore throat and tonsillitis as well as cervical lymphadenopathy despite multiple courses of antibiotics and a prednisone taper regimen. CT scans showed adenopathy of the bilateral cervical, bilateral axillary, mediastinal, abdominal and pelvic regions as well as splenomegaly. Biopsy of a right groin lymph node showed architectural effacement by a proliferation of small to intermediate sized cells with fine chromatin and scant cytoplasm (Fig. 1A). Immunohistochemical stains and flow cytometric studies showed positivity for CD1a, CD3, CD4 (variable), CD5, CD7, CD33, CD99, and TdT. The neoplastic cells were negative for CD8, CD10, CD34 and myeloperoxidase. A diagnosis of T lymphoblastic leukemia was rendered. The patient’s cerebrospinal fluid was negative for leukemia. A scrotal ultrasound revealed no intratesticular mass.

CBC evaluation showed a marked leukocytosis (98.71 k/uL) with 69% neutrophils, 3% metamyelocytes, 1% myelocytes, 11% lymphocytes, 16% monocytes, 0% eosinophils, and 0% basophils. Hemoglobin levels were normal (14.4 g/dL) and there was slight thrombocytopenia (platelets 140 k/uL). Circulating blasts were not identified. Bone marrow biopsy showed a markedly hypercellular bone marrow (> 95% cellular) with trilineage hematopoiesis, marked myeloid predominance (myeloid:erythroid ratio = 42), mild reticulin fibrosis, and 3% blasts (Fig. 1B). Flow cytometric studies and immunohistochemical stains showed the blasts to have an unremarkable myeloid phenotype with no evidence of a T lymphoblastic population.

**Fig. 2:**
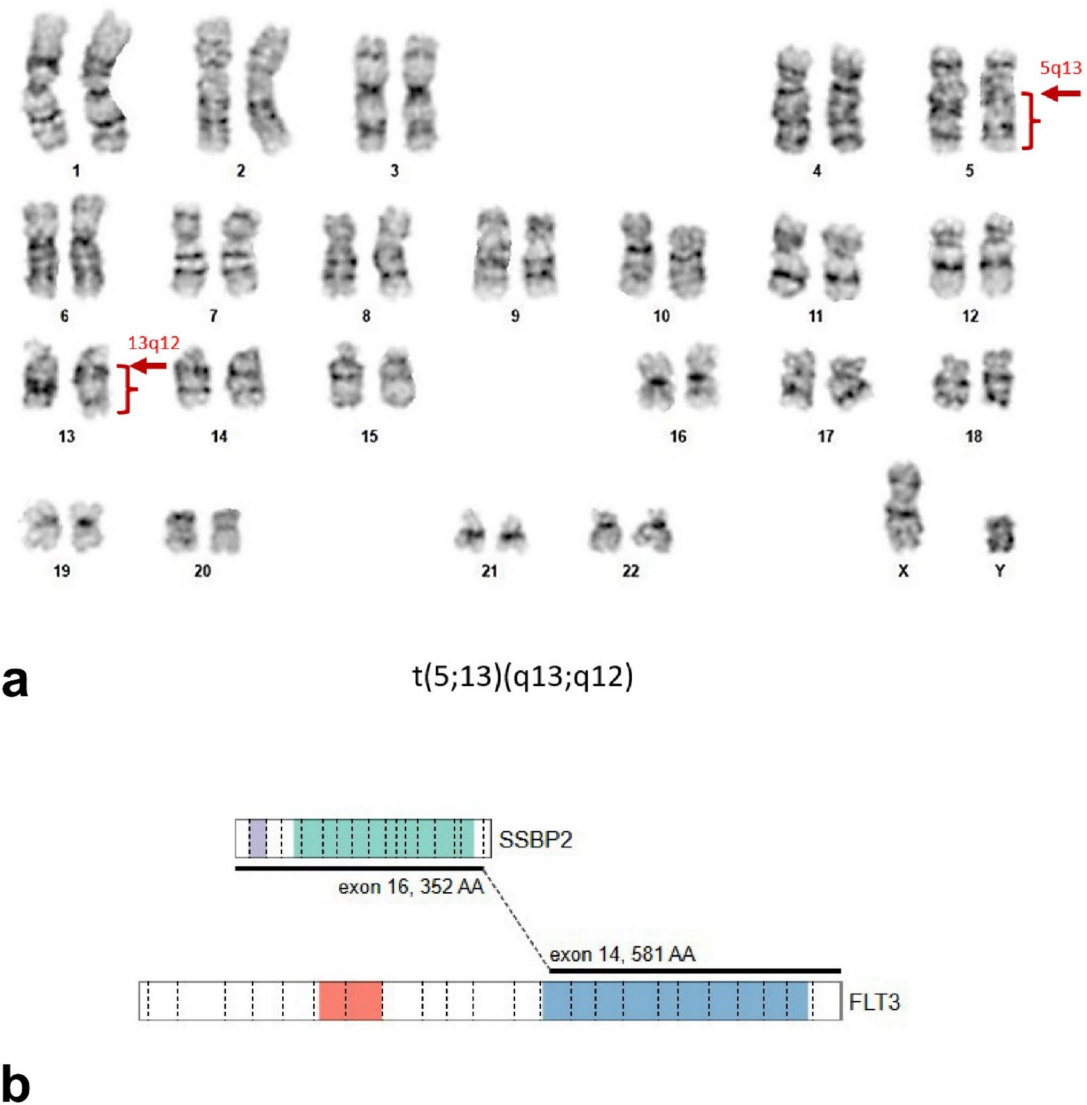
**A** Metaphase cytogenetic studies of the bone marrow demonstrated an abnormal karyotype with t(5;13)(q13;q12). **B** Schematic of *SSBP2::FLT3* fusion gene. The colored regions represent the lissencephaly type-1-like homology motif (gray) and single-stranded DNA binding protein domain (green) of *SSBP2* (NM_001256732.2) and the immunoglobulin (red) and tyrosine kinase domains (blue) of *FLT3* (NM_004119.2)

Metaphase cytogenetic studies of the bone marrow demonstrated an abnormal karyotype: 46,XY,t(5;13)(q13;q12)[16]/46,XY[4] (Fig. 2A). DNA based NGS studies with a 63 gene panel showed no pathologic variants, but an anchored multiplex amplicon-based RNA NGS assay using a 107 gene panel identified an in-frame *SSBP2::FLT3* fusion between *SSBP2* exon 16 and *FLT3* exon 14. The resulting fusion gene is predicted to join the nearly full-length *SSBP2* transcript together with the tyrosine kinase domains of *FLT3* (Fig. 2B). A final diagnosis of M/LN-eo with *FLT3* rearrangement was established.

The patient was treated per CALGB 10403 with the addition of nelarabine per COG AALL0434. His end of induction evaluation showed only a partial response with persistence of t(5;13) on bone marrow assessment and incomplete resolution of lymphadenopathy on CT scans. Due to worsening inguinal lymphadenopathy during consolidation, gilteritinib was added to his regimen based on prior success of FLT3 targeting in T-LBL for patients with identified *FLT3* re-arrangements [11]. Follow-up PET CT scan at end of consolidation was negative for FDG avid neoplastic uptake. The patient underwent a myeloablative matched unrelated donor allogeneic bone marrow transplant. His transplant conditioning regimen included cyclophosphamide and total body irradiation (1200 cGy) and the graft-vs.-host disease prophylaxis consisted of tacrolimus, mycophenolate mofetil and methotrexate. A bone marrow biopsy performed on post-transplant day 95 showed no morphologic evidence of leukemia, and metaphase cytogenetic studies and MRD flow cytometry studies were negative. Serial follow-up PET/CT scans have confirmed that the patient has remained in complete remission two years post-transplant. He has been off immunosuppressant therapy without evidence of graft-versus-host disease and has had no infections or other complications.

## Discussion

M/LN-eo-FLT3 is a rare diagnosis, with fewer than 40 cases reported in the literature to date [3, 10, 11]. The most frequent fusion gene is t(12;13)(p13.2;q12) *ETV6::FLT3*, representing approximately half of reported cases [1, 2]. Other reported partner genes include *SPTBN1* (2p16.2), *GOLGB1* (3q13.33), *ZYMY2* (13q12.11), *CCDC88C* (14q32.11), *TRIP11* (14q32.12), *MYO18A* (17q11.2) and *BCR* (22q11.23). To the best of our knowledge, the current case represents the first report of a novel t(5;13)(q13;q12) *SSBP2::FLT3* fusion. The structure of the predicted fusion gene is similar to other *FLT3* fusions, with the translocation breakpoint located in *FLT3* exon 14 such that the entire tyrosine kinase domain is included in the resulting fusion protein. Fusions of *SSBP2*, a tumor suppressor gene that appears to play a role in regulating hematopoietic differentiation, with other tyrosine kinase genes have been previously reported, including *SSBP2::CSF1R* [12] and *SSBP2::JAK2* [13] in B lymphoblastic leukemia and *SSBP2::FER* in T lymphoblastic leukemia [14]. Other partners include *NTRK1* (1q23.1), *SERINC5* (5q14.1), *WIPF3* (7p14.3), and *LGALS2* (22q13.1) [15]. In this case, the lack of other mutations identified on DNA- and RNA-based sequencing suggests that the *SSBP2::FLT3* fusion is a primary oncogenic driver in this disease.

The heterogeneous clinical presentations and morphologic features in M/LN-eo-TK present numerous challenges in routine diagnosis. Despite the formal name of this family of entities, up to one third of cases of M/LN-eo-TK lack peripheral blood eosinophilia. Timely identification of the underlying fusion gene is important for appropriate clinical management as many of these fusion genes impart sensitivity to targeted therapeutic regimens. Metaphase cytogenetic studies alone are insufficient for definitive identification of these abnormalities as several recurrent fusion genes are known to be cryptic by banded karyotyping. While FISH studies are suitable for detecting many of these rearrangements, the growing number of critical TK genes necessitates a large panel of probes that may not be feasible or economical. Increasingly, RNA-based NGS methods, either as targeted fusion panels or as a component of whole transcriptome analysis, can be used to efficiently screen for these pathogenic fusion genes. Clinicians and pathologists should have a low index of suspicion for a M/LN-eo-TK when evaluating patients with and without unexplained eosinophilia in the context of extramedullary lymphoid or myeloid disease or unusual myeloproliferative disorders.

## Data Availability

No datasets were generated or analysed during the current study.

## References

[1] Saft L, Kvasnicka HM, Boudova L, Gianelli U, Lazzi S, Rozman M (2023) Myeloid/lymphoid neoplasms with eosinophilia and tyrosine kinase fusion genes: a workshop report with focus on novel entities and a literature review including paediatric cases. Histopathology 83(6):829–849 37551450 10.1111/his.15021

[2] Tzankov A, Reichard KK, Hasserjian RP, Arber DA, Orazi A, Wang SA (2023) Updates on eosinophilic disorders. Virchows Arch 482(1):85–97 36068374 10.1007/s00428-022-03402-8

[3] Wang SA, Orazi A, Gotlib J, Reiter A, Tzankov A, Hasserjian RP, Arber DA, Tefferi A (2023) The international consensus classification of eosinophilic disorders and systemic mastocytosis. Am J Hematol 98(8):1286–1306 37283522 10.1002/ajh.26966

[4] WHO (2008) classification of tumours of haematopoietic and lymphoid tissues., 4th ed., IARC Press, Lyon

[5] WHO (2017) classification of tumours of haematopoietic and lymphoid tissues, IARC Press, Lyon

[6] Arber DA, Borowitz MJ, Cook JR, De Leval L, Goodlad JR, Hasserjian RP, King RL, Kvasnicka HM, Orazi A (2025) The international consensus classification of myeloid and lymphoid neoplasms. Wolters Kluwer, Philadelphia, PA, p 832

[7] Haematolymphoid Tumours (2024) 5th ed., International Agency for Research on Cancer, Lyon

[8] Pozdnyakova O, Orazi A, Kelemen K, King R, Reichard KK, Craig FE, Quintanilla-Martinez L, Rimsza L, George TI, Horny HP, Wang SA (2021) Myeloid/lymphoid neoplasms associated with eosinophilia and rearrangements of PDGFRA, PDGFRB, or FGFR1 or with PCM1-JAK2. Am J Clin Pathol 155(2):160–178 33367495 10.1093/ajcp/aqaa208

[9] Schoelinck J, Gervasoni J, Guillermin Y, Beillard E, Pissaloux D, Chassagne-Clement C (2024) T cell phenotype and lack of eosinophilia are not uncommon in extramedullary myeloid/lymphoid neoplasms with ETV6::FLT3 fusion: a case report and review of the literature. Virchows Arch 484(5):853–857 37985498 10.1007/s00428-023-03693-5

[10] Reiter A, Metzgeroth G, Cross NCP (2025) How I diagnose and treat myeloid/lymphoid neoplasms with tyrosine kinase gene fusions. Blood 145(16):1758–1768 39046810 10.1182/blood.2023022417

[11] Tang G, Tam W, Short NJ, Bose P, Wu D, Hurwitz SN, Bagg A, Rogers HJ, Hsi ED, Quesada AE, Wang W, Miranda RN, Bueso-Ramos CE, Medeiros LJ, Nardi V, Hasserjian RP, Arber DA, Orazi A, Foucar K, Wang SA (2021) Myeloid/lymphoid neoplasms with FLT3 rearrangement. Mod Pathol 34(9):1673–1685 33990705 10.1038/s41379-021-00817-7

[12] Schwab C, Roberts K, Boer JM, Gohring G, Steinemann D, Vora A, Macartney C, Hough R, Thorn Z, Dillon R, Escherich G, Cazzaniga G, Schlegelberger B, Loh M, den Boer ML, Moorman AV, Harrison CJ (2021) SSBP2-CSF1R is a recurrent fusion in B-lineage acute lymphoblastic leukemia with diverse genetic presentation and variable outcome. Blood 137(13):1835–1838 33197935 10.1182/blood.2020008536

[13] Poitras JL, Dal Cin P, Aster JC, Deangelo DJ, Morton CC (2008) Novel SSBP2-JAK2 fusion gene resulting from a t(5;9)(q14.1;p24.1) in pre-B acute lymphocytic leukemia. Genes Chromosomes Cancer 47(10):884–889 18618714 10.1002/gcc.20585

[14] Atak ZK, Gianfelici V, Hulselmans G, De Keersmaecker K, Devasia AG, Geerdens E, Mentens N, Chiaretti S, Durinck K, Uyttebroeck A, Vandenberghe P, Wlodarska I, Cloos J, Foa R, Speleman F, Cools J, Aerts S (2013) Comprehensive analysis of transcriptome variation uncovers known and novel driver events in T-cell acute lymphoblastic leukemia. PLoS Genet 9(12):e1003997 24367274 10.1371/journal.pgen.1003997 PMC3868543

[15] Dessen P (2025) SSBP2 (Single Stranded DNA Binding Protein 2). Accessed 8/1/2025. http://atlasgeneticsoncology.org/gene/46000/ssbp2-(single-stranded-dna-binding-protein-2

